# Farm characteristics and management routines related to neonatal porcine diarrhoea: a survey among Swedish piglet producers

**DOI:** 10.1186/s13028-016-0261-0

**Published:** 2016-11-10

**Authors:** Jenny Larsson, Nils Fall, Maria Lindberg, Magdalena Jacobson

**Affiliations:** 1 Department of Clinical Sciences, Swedish University of Agricultural Sciences, Box 7054, SE-75007 Uppsala, Sweden; 2 Farm and Animal Health, Kungsängens Gård hus 6 B, SE-753 23 Uppsala, Sweden

**Keywords:** New neonatal porcine diarrhoea, Neonatal porcine diarrhoea, Prevalence, Risk factors, Pigs, Swine, Treatment, Prevention

## Abstract

**Background:**

In recent years reports from a number of countries, including Sweden, describe problems with diarrhoea in newborn piglets despite the use of previously effective preventive measures. This seemingly altered disease pattern of neonatal porcine diarrhoea (NPD) warrants investigations on the magnitude and manifestation of the problem. The aim of the present study was to investigate the herd-level prevalence of NPD in Sweden and to describe disease characteristics and intervention strategies used in affected herds. To obtain this information a questionnaire was developed and sent out to 170 randomly selected herds. The presence of NPD in the herds was specified as “Yes”, “No” or “Occasional cases” during the preceding year.

**Results:**

A response rate of 58% (98/170) was achieved. The total prevalence of farmer experienced NPD, including occasional cases was 79.6% (95% CI 70.6–86.4%). Most herds (85%; 83/98) employed maternal vaccination against enterotoxigenic *Escherichia coli* (ETEC). The most common treatment regimens used in affected herds included antimicrobials only (43%; 18/42) or antimicrobials in combination with supplementary fluids (33%; 14/42). Trimethoprim in combination with a sulphonamide was the drug of choice in 57% (24/42) of the affected herds whereas the remaining herds used a broad range of other antimicrobials (neomycin, amoxicillin, fluoroquinolones, penicillin, and tylosin). Furthermore, the risk of experiencing NPD was found to be higher in herds with >200 sows (OR = 4.0) compared to herds with <200 sows and in herds where more ambitious efforts (such as providing supplemental colostrum or practicing split-suckling) were made to save weak-born piglets (OR = 4.4).

**Conclusions:**

The results of the present study indicate that Swedish farmers commonly experience NPD in their herds, often despite vaccination against ETEC. Considering the extent of this problem and its contribution to antimicrobial usage, improving alternative control strategies for NPD needs to be prioritized.

**Electronic supplementary material:**

The online version of this article (doi:10.1186/s13028-016-0261-0) contains supplementary material, which is available to authorized users.

## Background

Neonatal porcine diarrhoea (NPD) is a common and serious problem causing significant production losses in pig farming worldwide [1–3]. A number of infectious agents have been associated with NPD, however the occurrence of diarrhoea in newborn piglets should be viewed as the result of a multitude of interacting factors [4]. Major determinants for the manifestation of NPD are for instance the infection pressure exerted by specific pathogens present in the herd, the environmental temperature, and the passive immunity transferred by colostrum [4–8]. Many management practices and herd characteristics may hence be associated with either an increased or decreased risk of disease [9–14].

In Sweden, well-known causes of NPD include enterotoxigenic *Escherichia coli* (ETEC) and *Clostridium perfringens* type C, two agents that can be prevented by maternal vaccination combined with a high level of hygiene in the farrowing units [1, 15, 16]. In recent years however, reports from a number of countries, including Sweden, describe problems with NPD despite the use of previously effective preventive measures [17–20]. The underlying causes for this problem, by some referred to as new neonatal porcine diarrhoea syndrome (NNPDS), is currently unknown and hitherto well-known porcine enteropathogens seem to be of minor importance [17, 19, 20].

This seemingly altered disease pattern of NPD warrants further investigations on the magnitude and manifestation of the problem. The aim of the present study was hence to investigate the prevalence of NPD in Sweden and to describe disease characteristics and intervention strategies used in affected herds. Furthermore, we aimed to identify herd characteristics and herd management practices that differentiate herds experiencing a problem with neonatal porcine diarrhoea from herds not experiencing NPD.

## Methods

### Study design and data collection

A questionnaire was developed to obtain information on farm characteristics, management routines, and, in affected herds, disease manifestation and intervention strategies. The questionnaire was designed to be filled in by the farmers assisted by their veterinarian, and was pilot-tested in six piglet-producing herds to improve the comprehensiveness of the questions. These responses were not included in subsequent analysis.

The study population was piglet-producing herds supervised by the Swedish Animal Health Service (presently known as Farm and Animal Health) or by Lunden’s Animal Health Service. In 2011, 740 piglet-producing herds (all production types including sows) were supervised by the Swedish Animal Health Service, and 59 by Lunden’s Animal Health Service (personal communication, S.O. Dimander, Swedish Animal Health Service, and E. Lindahl, Lunden’s Animal Health Service). This corresponded to approximately 86% of the total number of herds keeping sows in Sweden during 2011 [21]. We hypothesised that a higher response rate would be achieved if the questionnaires were distributed by the veterinarians at their routine herd visits, compared to distributing the questionnaires per mail.

In agreement with the 17 veterinarians participating in the study, an average of ten questionnaires was assigned per veterinarian. The sample size of 170 was hence indirectly decided by the study design. The random selection of herds (from the 799 in total) was however not stratified and resulted in four to 16 herds per veterinarian. Herds that had less than ten sows or had ceased their piglet production were replaced.

The final questionnaire included 59 questions divided into two sections—“production and management routines”, and “disease characteristics and treatment of neonatal diarrhoea”. The section on production and management covered type of production, age of farrowing stables, number of sows in production, number of employees, recording of production data and production results, employment of all in-all out in the farrowing unit, washing and disinfection of the farrowing unit, vaccination routines against NPD, employment of nurse sows, entry of sows to the farrowing unit, treatment of post–partum dysgalactia syndrome (PPDS), monitoring of farrowing, and management of newborn piglets and their environment. The section on disease characteristics and treatment of NPD in affected herds included proportion of piglets affected, changes in prevalence of NPD, prevalence in litters from first parity sows, time point for the occurrence of diarrhoea, treatment of diarrhoea, efforts made to investigate the cause, and employment of prophylactic measures.

The questionnaire design included multiple choice or dichotomous questions as well as a smaller number of open-ended or semi-open questions. Respondents were requested to base their answers on an average farrowing batch during the preceding year. The full questionnaire (in Swedish) is available upon request from the corresponding author.

Questionnaires and a stamped, addressed return envelope, as well as a short missive with instructions to the veterinarians, were distributed by mail during February 2012 to the 16 veterinarians employed by the Swedish Animal Health Service. A reminder to the veterinarians was sent out in May. In November, a second reminder was sent out to individual veterinarians by e-mail together with a list of herds from which responses were missing. Three veterinarians withdrew from the study. Following an initial telephone contact, these questionnaires were instead distributed to the corresponding herds by mail. A similar procedure was employed for herds supervised by Lunden’s Animal Health Service (n = 11).

### Data editing

**Fig. 1:**
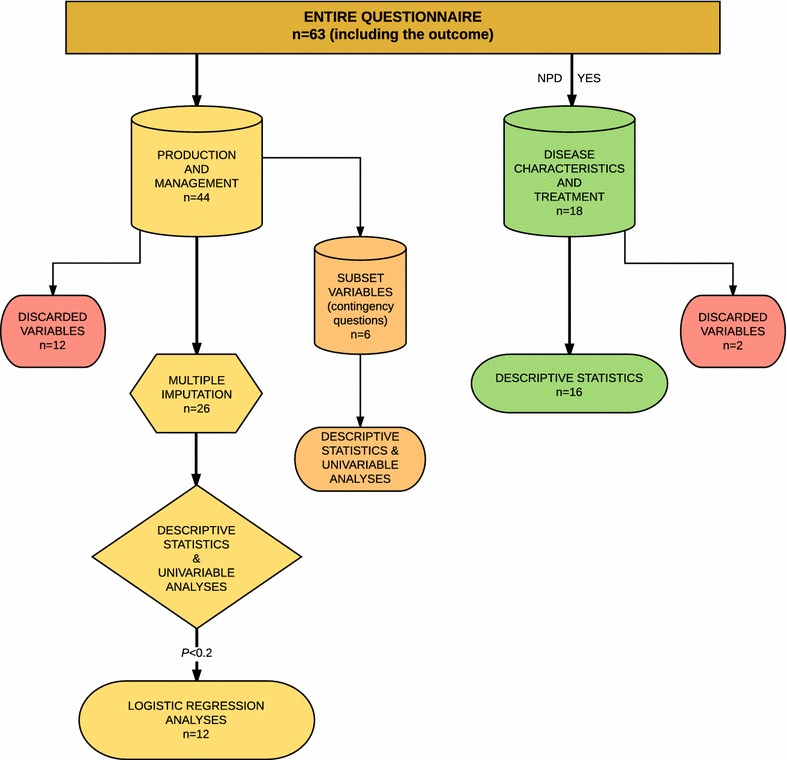
Overview of data processing and analyses. The questionnaire included a total of 59 questions corresponding to 63 variables (including the outcome), as some questions included information on more than one parameter. In the illustration above “n” denotes the number of variables in the different sections of the questionnaire and in the following data processing and statistical analyses

In total, the 59 questions in the questionnaire corresponded to 63 different variables (including the outcome) as some questions included information on more than one parameter. Due to poor question design or low response rates (>25% missingness), 14 questions were discarded (10 and 4, respectively). See Fig. 1 for an overview.

All answers were treated as categorical variables. To facilitate the analyses, some answers were amalgamated into fewer categories. Answers on interval scale was categorised in quartiles (“sow batch size”). Other semi-open questions with numerical answers were often given in intervals, e.g. 2–3, and were categorised accordingly (“entry of sows to the farrowing unit” and “desired temperature in the farrowing unit”). The outcome “presence of neonatal diarrhoea”, was specified in the questionnaire as diarrhoea in piglets younger than seven days with the possible responses “Yes”, “No”, and “Occasional cases”. In the analyses, the outcome was treated as a binomial variable where “No” and “Occasional cases” were merged.

Most variables (Tables 1, 2, 3) are self-explanatory but some clarifications may be needed. “Efforts made to save weak-born piglets” were categorized as “none/some efforts” and “moderate/ambitious efforts” Criteria for categorising the answers as “some” were: one or two simple measures such as placing the piglet under the heat lamp or by the udder. Criteria for categorising the answers as “moderate/ambitious” were: two or more simple measures as stated above or providing supplemental colostrum, providing active heating of piglets such as placement in warm water, or practicing split-suckling. Vaccination routines were categorised based on compliance with the current recommendations. For questions related to production, only data from respondents systematically recording data using PigWin Sugg [22] were included. From these production parameters, “number of live born piglets/sow” and “number of weaned piglets/sow” were selected and the answers compared with the national averages (includes data from 58 657 sows from 149 Swedish herds) of 13.2 and 10.8, respectively [23]. The answers were categorized as “good”: both parameters above average, or one parameter above and one on average, “intermediate”: one parameter above average and one below, or “low”: both parameters below average, or one on average and one below.

**Table 1:** Distribution and univariable analysis of herd characteristics in 42 herds experiencing neonatal porcine diarrhoea (NPD herds) and 56 herds with no or occasional cases (non-NPD herds)

Variable^a^	NPD herds n (%)	Non-NPD herds n (%)	*P* value^b^
*Building or renovation of stables used as farrowing units*			0.05^d^
Earlier than 1990	3 (7)	11 (20)	
1990–2000	7 (17)	18 (32)	
Varies between units	12 (29)	10 (18)	
Later than 2000	20 (48)	17 (30)	
*Number of sows in production*			<0.01^c^
<200	20 (48)	44 (79)	
>200	22 (52)	12 (21)	
*Batch size*			<0.01^c^
<20	6 (14)	23 (41)	
20–34	9 (21)	16 (29)	
>34–46	12 (29)	11 (20)	
>46	15 (36)	6 (11)	
*Recording of production results*			0.02^d^
Yes	40 (95)	44 (79)	
No	2 (5)	12 (21)	
*Level of gilt recruitment*			0.18^c^
<30%	9 (21)	18 (32)	
30–40%	10 (24)	19 (34)	
>40%	16 (39)	11 (20)	
Unknown	7 (17)	8 (14)	

^a^Respondents were requested to base their answers on an average farrowing batch during the last 12 months

^b^P value of the entire variable

^c^χ^2^-test

^d^Fisher’s exact test

**Table 2:** Distribution and univariable analysis of management routines in 42 herds experiencing neonatal porcine diarrhoea (NPD herds) and 56 herds with no or occasional cases (Non-NPD herds)

Variable^a^	NPD herds n (%)	Non-NPD herds n (%)	*P* value^*b*^
*Manual cleaning of the farrowing unit (times/day)* ^*c*^			0.12^d^
0–1	27 (64)	26 (46)	
2–3	15 (36)	30 (58)	
*Washing of the farrowing unit between batches*			<0.01^e^
Always	35 (83)	32 (57)	
During summer	5 (12)	9 (16)	
No	2 (5)	15 (27)	
*Disinfection of the farrowing unit between batches*			0.07^d^
Yes	28 (67)	26 (46)	
No	14 (33)	30 (54)	
*Maternal vaccination against NPD*			0.02^e^
Yes	40 (95)	43 (83)	
No	2 (5)	13 (25)	
*Employment of nurse sows*			0.01^d^
Yes	24 (57)	17 (30)	
No	18 (43)	39 (70)	
*Monitoring of farrowings*			0.17^e^
Only if indicated	3 (7)	11 (20)	
During daytime	35 (83)	38 (68)	
During day and night	4 (10)	7 (13)	
*Efforts made to save weak*-*born piglets*			<0.01^d^
None/some	18 (43)	43 (77)	
Moderate/ambitious	24 (57)	13 (23)	
*Type of supplemental heating in the creep area*			0.19^e^
Heat lamp	11 (26)	24 (43)	
Floor heating	4 (10)	6 (10)	
Lamp and floor heating	27 (64)	25 (45)	
Other	0 (0)	1 (2)	

^a^Respondents were requested to base their answers on an average farrowing batch during the last 12 months

^b^P value of the entire variable

^c^First week after farrowing

^d^χ^2^-test

^e^Fisher’s exact test

**Table 3:** Disease characteristics and management of neonatal porcine diarrhoea (NPD) in 42 herds experiencing more than occasional NPD-cases

Variable	Responses
	n (%)
Age of affected piglets	
First 24 h	2 (5)
1–3 days	26 (62)
3–7 days	9 (21)
Varied	3 (7)
Missing	2 (5)
Higher prevalence of NPD in litters from first parity sows	
Yes	34 (81)
No	7 (17)
Not known	1 (2)
Treatment of NPD	
Antimicrobials	18 (43)
Antimicrobials and fluids	14 (33)
Antimicrobials and fluids, and NSAID	2 (5)
”Injection”	3 (7)
Prophylactic treatment with antimicrobials	1 (2)
Missing	4 (10)
First choice of antimicrobial to treat NPD	
Amoxicillin	5 (12)
Penicillin	2 (5)
Fluoroquinolones	3 (7)
Neomycin	6 (14)
Trimethoprim/sulphonamide (sulfadiazine or sulfadoxine)	24 (57)
Tylosin	1 (2)
Missing	1 (2)
Prophylactic measures used against NPD	
Changed vaccination routines	5 (12)
Hygienic measures	6 (14)
Vaccination	2 (5)
Vaccination and hygienic measures	2 (5)
Faecal feedback	6 (14)
No specific measures	20 (48)
Missing	1 (2)

### Statistics

Prior to statistical analyses, missing values for explanatory variables in the section “production and management routines” were managed by multiple imputation (MI) using a nonparametric method in the CRAN package MissForest [24, 25]. In MI, missing values are predicted based on the observed values for other variables in the dataset. The imputed dataset hence maintains the overall variability in the population while preserving relationships with other variables. The dataset for imputation only contained variables applicable for all respondents and hence, answers to contingency questions corresponding to six variables were removed before imputation. The dataset therefore totalled 26 explanatory variables for which the maximum missingness of any individual variable was 10% (Fig. 1).

The associations between the explanatory variables and NPD were initially analysed univariably (χ^2^ –test or Fisher’s exact tests in case of cell frequencies <5). The results were compared with equivalent analyses on the original non-imputed data with no considerable differences (see Additional file 1: Table S1). Variables with an association to NPD of *P* < 0.2 in the univariable analyses were subsequently examined by logistic regression models. The variable “number of sows per farrowing batch” was, however, excluded as sows in production (SIP) was considered a better measure of herd size. Hence, a total of 12 explanatory variables were included (Tables 1, 2). To check the selected explanatory variables for multicollinearity, variance inflation factors (VIF) were calculated using the CRAN package car [26], interpreting a VIF >5 as indicative for multicollinearity. No variable was removed due to multicollinearity. As herd size may be assumed to be associated with differences in production and management routines, the relationship between these variables and SIP was evaluated. Out of the 11 variables, all but “disinfection between farrowing batches” and “monitoring of farrowings” were significantly associated with SIP in univariable tests (see Additional file 2: Table S2 for descriptive data and univariable analyses). Due to these inter variable relationships the effect of SIP was estimated in a univariable logistic regression model whereas the other variables were analysed in a multivariable logistic regression model. Multivariable model building was done by backward stepwise elimination with re-entering based on Wald-tests of main effects until all remaining effects were significant at *P* < 0.05. Potential confounding was considered in each step by comparing the estimates of the variables with and without the possible confounder. A change of >30% in the estimates was interpreted as confounding, in which case the variable was retained in the model.

Apart from variables included in the imputed dataset, descriptive statistics and univariable analysis of associations with NPD are presented for subset variables from the sections “production and management”. Variables from the section “disease characteristics and treatment of NPD in affected herds” are presented descriptively. All statistical analyses were performed in R version 3.2.2 [27].

## Results

Of the 101 questionnaires returned by April 2013 one was blank. Further, information on the presence of NPD in the herd was missing from two respondents. The number of usable questionnaires therefore totalled 98 (response rate of 58%). Forty-two herds reported experience of more than occasional cases of NPD during the previous year, 36 reported occasional cases and 20 reported no occurrence. Hence, the total prevalence of farmer experienced NPD, including occasional cases was 79.6% (95% CI 70.6–86.4%) whereas the prevalence of herds experiencing a recurrent problem with NPD was 43% (95% CI 33.5–52.7%).

### Herd characteristics and management routines

Of the 98 herds included in the study, 43 had integrated or partially integrated production, 34 were specialised piglet producers, 18 were satellite herds in sow pool-systems, and three were specialised gilt-producing or nucleus herds. The majority of herds (n = 64) had less than 200 sows in production. Of the 63 herds that recorded production results with PigWin Sugg, 23 had production results that were classified as “good”, 10 had “intermediate” results, and 21 had production results that was lower than average (information was lacking from 9 herds). There was no statistical difference between NPD and non-NPD herds (*P* = 0.68).

All in-all out was practiced in the farrowing unit in 86 of the 98 herds and 67 washed the farrowing units between each batch. Maternal vaccination against agents associated with NPD was routinely performed in 83 of the herds. All vaccinating herds used vaccines against ETEC, seven in combination with *C. perfringens* type C and three in combination with *C. perfringens* type A and C (information was lacking from six herds). Eleven herds had changed vaccine during the last year. Most herds vaccinated sows and gilts at least three weeks prior to farrowing (68 and 52 out of the 83, respectively). However, in ten herds (four NPD and six non-NPD herds), gilts were only vaccinated once. Supplemental heating of the creep area was provided in all herds. However, in herds using heat lamps as the only heat source (n = 34), lamps with a low wattage (≤150 W) were more common in herds with NPD (*P* = 0.02).

Herd characteristics and management routines with associations to NPD of *P* < 0.2 in univariable tests are presented in Tables 1 and 2, respectively. Data on additional variables applicable for all 98 herds are available in Additional file 1: Table S1.

### Analysis of potential risk factors for neonatal porcine diarrhoea on herd level

The univariable logistic regression analysis on the effect of herd size (number of sows in production, SIP) on the presence of NPD showed that herds with >200 SIP had a higher risk of having NPD compared with herds <200 SIP (*P* < 0.01). The estimated odds ratio for herds with >200 SIP was 4.0 (95% CI 1.7–10).

The multivariable logistic regression analysis of management factors rendered a final model which only included the variable “efforts made to save weak-born piglets” (*P* < 0.01). Herds where moderate to ambitious efforts were made to take care of weak piglets had a higher risk of having NPD compared with herds where none or only some efforts were made. The estimated odds ratio for herds where moderate to ambitious efforts were made was 4.4 (95% CI 1.9–11).

### Disease characteristics and treatment of NPD

Among the 42 herds with more than occasional cases of NPD, the majority (33 out of 42) estimated that less than 25% of the piglets were affected by diarrhoea in an average farrowing batch. Diarrhoea was most common 1–3 days after birth (26 out of 42) and did often affect more than 50% of piglets in diarrhoeic litters (29 out of 42). The mortality due to NPD was estimated to be <10% in most herds (30 out of 42). In 29 of the 42 herds, the occurrence of diarrhoea in the herd was described as constant (18) or intermittent (11), whereas ten stated that the diarrhoea had decreased, and three that it had increased during the last year. Seasonal variations were only described in four herds (more during winter and spring). Diarrhoeic piglets were most commonly treated with antimicrobials only, or antimicrobials in combination with supplemental fluids (18 out of 42 and 14 out of 42, respectively). The drug of choice was trimethoprim/sulphonamide (24 out of 42) and 34 stated that treatment was successful in more than 75% of the cases. In case of poor response to initial treatment, 19 stated that they used fluoroquinolones as a second drug of choice. Efforts to investigate the cause of NPD had been undertaken in 13 out of 42 herds during the preceding year and three stated that the cause of diarrhoea had been established.

The prophylactic measures used to prevent NPD were grouped as “changed vaccination routines” (5), “hygienic measures” (6), “vaccination” (2), “vaccination and hygienic measures” (2), “faecal feedback” (6), and “no specific measures” (20). Hygienic measures were often not specified, and although most herds with diarrhoea vaccinated against NPD, only two respondents mentioned vaccination alone as a specific prophylactic measure. Among the 21 respondents reporting that specific prophylactic measures were employed, the effect was appreciated to be poor by two, moderate by 12, and successful by five (three missing answers). Measures with successful effect were hygienic measures and/or vaccination. Faecal back feeding was estimated to have poor (2) or moderate effect (4). An overview of disease characteristics and treatment of NPD in affected herds are presented in Table 3.

## Discussion

The total herd-level prevalence of farmer experienced NPD (79.6%; 95% CI 70.6–86.4%) indicates that diarrhoea in neonatal piglets is common among Swedish piglet-producing herds. Interestingly, many herds experienced more than occasional NPD cases, despite maternal vaccination against ETEC or ETEC and *C. perfringens* (93% of the 42 herds with more than occasional cases of NPD). Moreover, a broad range of different antimicrobials were used for treatment (trimethoprim/sulphonamide, neomycin, amoxicillin, fluoroquinolones, penicillin, and tylosin). Taken together, these results suggest that NPD is a substantial problem and that prophylaxis and treatment regimens recommended against ETEC are not always efficient. Further, the results indicated that larger herd size (>200 SIP) and making more efforts to save weak-born piglets could be potential risk factors for experiencing NPD on herd-level.

### The estimated prevalence of diarrhoea on herd level

Previous studies on the herd-level prevalence of NPD are lacking and epidemiological studies on pre-weaning diarrhoea have hitherto focused on incidence rates on litter level [10, 11, 28, 29]. In the present study, presence of NPD in a herd was subjectively estimated by the individual farmer as “Yes”, “No”, or “Occasional cases” during the preceding year. Thus, the prevalence reported in this study estimates how Swedish farmers experienced the problem with NPD in their herds rather than being an objective measure of the occurrence of diarrhoea. Moreover, the reported “farmer experienced” herd-level prevalence of 79.6% (including occasional cases) may be influenced by selection bias since farmers from herds with NPD might have been more motivated to complete the questionnaire. However, even if all of the 72 non-responders were herds without NPD, the prevalence would still be considerable (45.9%, 95% CI 38.6–53.4%).

### Association of NPD with herd characteristics and management routines

To investigate whether presence of NPD on herd level could be associated with certain herd characteristics or management routines we wanted to compare herds experiencing a more recurrent problem with NPD from herds with less prominent or no problems. Thus, for these analyses the categories “occasional cases” and “no” were merged.

Before statistical analyses, missing data was handled by MI as MI analyses generally produce less biased and more precise (smaller standard errors) results than complete case analyses [30].

In the present study, larger herds (>200 SIP) were found to be significantly associated with experiencing NPD (OR = 4.0). The categorisation of herds size as <200 or >200 SIP was based on the average Swedish herd size of 186 sows [31]. Associations between herd size and risk for disease have previously been described for a number of infectious porcine diseases including pre-weaning diarrhoea [9, 11, 32]. The relationship has been discussed to not only depend on differences in the number of animals but also on environmental factors and management routines that may differ between smaller and larger herds [32]. This is supported by the present results where herd size was significantly associated with most of the herd characteristics and management routines that had a univariable association with NPD of *P* < 0.2 (see Additional file 2: Table S2). Assuming that these differences may partially explain the association between herd size and NPD, such factors could be considered as intervening variables. Statistical analysis of such interrelated variables is problematic. It is therefore recommended not to include intervening variables when investigating the association between explanatory variables (predictors) of interest and the outcome by multivariate modelling [33]. The association between herd size and NPD was hence analysed separately.

By multivariate analysis of other herd characteristics and management routines (n = 11), efforts made to save week-born piglets were shown to have a significant effect on the likelihood of experiencing NPD (*P* < 0.01). Interestingly, making more ambitious efforts to save weak piglets was associated with a higher risk of experiencing diarrhoea (OR = 4.4). One explanation for this may be that an increased handling of piglets could lead to an increased spread of pathogens between litters. However, it could also be that more ambitious animal caretakers were more likely to report NPD, or that newborn piglets were given extra attention because the herd had a NPD-problem. Lastly, it cannot be excluded that promoting survival of weak piglets indirectly could lead to an increased number of NPD-cases as these pigs may be more prone to develop diarrhoea.

Other factors significantly associated with NPD in the univariable tests were maternal vaccination against NPD, age of the farrowing building(s), recording of production results, washing of the farrowing unit, and employment of nurse sows (see Tables 1, 2). Maternal vaccination against ETEC was more common in herds with recurrent NPD as compared to herds with no or occasional cases (*P* = 0.02, see Table 2). As the presence of NPD is the incentive to vaccinate this is not surprising. However, the presence of more than occasional cases in vaccinating herds is notable (39 herds in total). Furthermore, 21% of the 39 vaccinating herds with NPD estimated that the average proportion of piglets affected per batch was >25%, thus indicating a substantial problem. The use of faecal back-feeding as a preventive measure against NPD in six of these herds further underlines that the effect of commercial vaccines was considered unsatisfactory. Possible explanations include inadequate vaccination routines, inadequate colostrum intake, or that piglets contracted diarrhoea despite a satisfactory passive immune status against ETEC and *C. perfringens* type C. Further, both the use of nurse sows and more rigorous washing routines (washing between each farrowing batch) were more common in herds experiencing more than occasional cases of NPD. These results hence follow the same trend as “efforts made to save weak-born piglets” suggesting that an increased use of zootechnical interventions could be associated with NPD. Again, it is however not possible to make any causal inferences from the present investigation. Nonetheless, a high level of intervention measures has previously been suggested as a contributing factor to recurrent NPD problems in high-performance herds [18, 34]. Thus, further investigations are needed.

Taken together, these results underline that the presence of NPD may be influenced by several aspects with complex interrelationships. It should however be stressed that the outcome in the present study reflects “farmer-experienced” NPD. Whereas this gives interesting descriptive information on how the presence of NPD is perceived, the subjective estimates of NPD-presence as “Yes”, “No”, or “Occasional cases” may not be consistent between herds. An objective classification of the presence of NPD should therefore be employed in future investigations of the potential risk factors suggested by the present study.

### Disease manifestation and intervention strategies

In accordance with previous studies, most farmers stated that diarrhoea was more common in first-parity sow litters [29, 35, 36]. This is probably a reflection of first-parity sows having a poorly developed immunity against pathogens present in the farrowing unit [37]. However, since the question was phrased in a way which may be considered leading (Is diarrhoea more common in litters from first parity sows?), the answers might have been skewed.

Treatment of NPD commonly included antimicrobials only (43%; 18/42) or combined with supplementary fluids (33%; 14/42). A smaller proportion (7%; 3/42) had only stated “by injection” which likely could be interpreted as injection with antimicrobials. Notably, microbiological investigations of NPD had only been undertaken in 31% (13/42) of the affected herds during the preceding year. In addition, one respondent stated that all piglets were treated with antimicrobials after birth to prevent NPD. This frequent use of antimicrobials against NPD makes the development of alternative control strategies a necessity. Highlighting this need, a study of antimicrobial usage in Swedish pig herds recently showed that the highest treatment incidence occurs in suckling piglets [38]. Targeted health improvements in this age group may therefore be the most efficient way to further decrease the use of antibiotics.

The drug of choice in the present investigation was trimethoprim in combination with sulfadiazine/sulfadoxine (57%; 24/42), which is the recommended treatment against ETEC. Interestingly, however, 40% (17/42) of the herds used other antimicrobials (one missing answer). This could suggest an unsatisfactory effect by trimethoprim/sulphonamide treatment due to resistant ETEC, or that the treatment targeted bacteria other than *E. coli* (e.g. the use of penicillin or tylosin). Since many herds also reported more than occasional cases of NPD despite ETEC vaccination, the results suggest that prophylaxis and treatment regimens recommended against ETEC not always are efficient. This corresponds to recent pathological and microbiological studies, demonstrating occurrence of NPD unrelated to the presence of previously well-known enteropathogens in Sweden and Denmark [17, 19]. Prevalence studies on enteropathogens associated with NPD would hence be of great interest.

### Considerations on the study design

The distribution of the questionnaire via the herd veterinarians was chosen as we hypothesised that this would yield a higher response rate as compared to distribution by mail. However, some veterinarians (4 out of 17) were not able to complete the study and thus, the corresponding questionnaires were instead distributed directly to the herds by mail. Due to the small number of questionnaires per veterinarian, differences between veterinarians and distribution method were however not included in the analysis and hence, these potential confounders should be kept in mind when interpreting the results. Furthermore, we do not know if responder and non-responder herds differed in terms of size, production results etc. since no data from non-responders were available. A nationwide, up-to-date register of pig herds in Sweden including information on herd size would greatly facilitate this type of studies. However, in the 63 herds using PigWin Sugg, production data was comparable to the average results reported by Swedish PigWin users during 2012.

Some information was not obtained due to inadequately designed questions (n = 10). Unfortunately, this included treatment frequencies of post-partum dysgalactia syndrome (PPDS) that is a known risk factor for NPD [10, 11, 28, 39]. The major reasons for the exclusion of questions were misinterpretation, or that the respondent had marked more than one response option making it impossible to create mutually exclusive categories. These problems could probably have been avoided by a more extensive pilot testing of the questionnaire. Moreover, one question excluded due to a low response rate (>25% missingness) was temperature in the creep area which possibly reflects that the temperature was not known by the respondents. This is noteworthy considering that the ambient temperature is known to be of great importance for the survival of the newborn pig [40–42].

## Conclusions

The results of the present study indicate that Swedish farmers commonly experience NPD in their herds, often despite vaccination against ETEC. Considering that antimicrobials are frequently used as treatment, preventive measures against NPD need to be improved. To find rational means for intervention, identification of factors potentially related to the disease is important. In the present study, larger herd size and making more ambitious efforts to save weak-born piglets were suggested as potential risk factors and further studies of the underlying biological reasons for these relationships are warranted.

## Additional files

**Additional file 1: Table S1.** Comparison of original and imputed data. Descriptive data and univariable associations between 26 variables and neonatal porcine diarrhoea in 98 herds (42 herds experiencing NPD and 56 experiencing no or only occasional cases) before and after multiple imputation of missing values for explanatory variables.

**Additional file 2: Table S2.** Associations with herd size. Descriptive data and univariable associations between herd size (<200 and >200 sows in production) and explanatory variables selected for regression analyses.
