# Supplementary material for: Farm characteristics and management routines related to neonatal porcine diarrhoea: a survey among Swedish piglet producers
Source: Acta Vet Scand. 2016 Nov 10;58:77. doi: 10.1186/s13028-016-0261-0 (PMC5103491; doi:10.1186/s13028-016-0261-0)
Supplement: Supplementary file 1 — Additional file 1: Table S1. Comparison of original and imputed data. Descriptive data and univariable associations between 26 variables and neonatal porcine diarrhoea in 98 herds (42 herds experiencing NPD and 56 experiencing no or only occasional cases) before and after multiple imputation of missing values for explanatory variables. [file 13028_2016_261_MOESM1_ESM.pdf]

Additional file 1. Descriptive data and univariable associations between 26 variables and neonatal porcine diarrhoea (NPD) in 42 herds experiencing NPD (NPD herds) and 56 experiencing no or only occasional cases (Non-NPD herds) before and after imputing data for missing answers.

	Original data			Imputed data			
**Variable [a]**	**NPD herds n (%)**	**Non-NPD herds n (%)**	**P-value**	**NPD herds n (%)**	**Non-NPD herds n (%)**	**P-value**	**Test**
**Production type:**			0.20			0.20	Fisher's exact
Integrated	13 (31)	17 (30)		13 (31)	17 (30)		
Partially integrated	7 (17)	6 (11)		7 (17)	6 (11)		
Specialised piglet production	10 (24)	24 (43)		10 (24)	24 (43)		
Satellite herds in sow pool systems	11 (26)	7 (13)		11 (26)	7 (13)		
Specialised gilt-producing or nucleus herds	1 (2)	2 (4)		1 (2)	2 (4)		
Missing	0 (0)	0 (0)					
**Building or renovation of stables used as farrowing units:**			0.05			0.05	Fisher's exact
Earlier than 1990	3 (7)	11 (20)		3 (7)	11 (20)		
1990-2000	7 (17)	18 (32)		7 (17)	18 (32)		
Varies between units	12 (29)	10 (18)		12 (29)	10 (18)		
Later than 2000	20 (48)	17 (30)		20 (48)	17 (30)		
Missing	0 (0)	0 (0)					
**Number of sows in production:**			<0.01			<0.01	$\chi 2$
<200	20 (48)	42 (75)		20 (48)	44 (79)		
>200	22 (52)	11 (20)		22 (52)	12 (21)		
Missing	0 (0)	3 (5)					

	Original data			Imputed data			
Variable [a]	NPD herds n (%)	Non-NPD herds n (%)	P-value	NPD herds n (%)	Non-NPD herds n (%)	P-value	Test
**Batch size:**			<0.01			<0.01	χ2
<20	5 (12)	20 (36)		6 (14)	23 (41)		
20-34	9 (21)	16 (29)		9 (21)	16 (29)		
>34-46	12 (29)	11 (20)		12 (29)	11 (20)		
>46	15 (36)	6 (11)		15 (36)	6 (11)		
Missing	1 (2)	3 (5)					
**Recording of production results:**			0.02			0.02	Fisher's exact
Yes	40 (95)	44 (79)		40 (95)	44 (79)		
No	2 (5)	12 (21)		2 (5)	12 (21)		
Missing	0 (0)	(0)					
**Level of gilt recruitment:**			0.42			0.18	χ2
<30%	8 (19)	15 (27)		9 (21)	18 (32)		
30-40%	10 (24)	17 (30)		10 (24)	19 (34)		
>40%	14 (33)	11 (20)		16 (39)	11 (20)		
Unknown	7 (17)	8 (14)		7 (17)	8 (14)		
Missing	3 (7)	5 (9)					
**Internal gilt recruitment:**			0.67			0.62	χ2
Yes	20 (48)	24 (43)		21 (50)	24 (43)		
No	17 (40)	27 (48)		21 (50)	32 (57)		
Missing	5 (12)	5 (9)					

	Original data			Imputed data			
Variable [a]	NPD herds n (%)	Non-NPD herds n (%)	P-value	NPD herds n (%)	Non-NPD herds n (%)	P-value	Test
**Health status monitoring in the farrowing unit (times/day)[b]:**			0.37			0.35	χ2
1-2	5 (12)	13 (23)		5 (12)	13 (23)		
3-4	12 (29)	13 (32)		12 (29)	13 (32)		
>4	24 (57)	30 (54)		25 (60)	30 (54)		
Missing	1 (2)	0 (0)					
**Manual cleaning of the farrowing unit (times/day)[b]:**			0.21			0.12	χ2
0-1	25 (60)	26 (46)		27 (64)	26 (46)		
2-3	15 (36)	29 (52)		15 (36)	30 (58)		
Missing	2 (5)	1 (2)					
**Emptying of the farrowing unit between batches:**			0.22			0.22	Fisher's exact
Yes	39 (93)	47 (84)		39 (93)	47 (84)		
No	3 (7)	9 (16)		3 (7)	9 (16)		
Missing	0 (0)	(0)					
**Washing of the farrowing unit between batches:**			<0.01			<0.01	Fisher's exact
Always	35 (83)	32 (57)		35 (83)	32 (57)		
During summer	5 (12)	9 (16)		5 (12)	9 (16)		
No	2 (5)	15 (27)		2 (5)	15 (27)		
Missing	0 (0)	(0)					

	Original data			Imputed data			
Variable [a]	NPD herds n (%)	Non-NPD herds n (%)	P-value	NPD herds n (%)	Non-NPD herds n (%)	P-value	Test
**Method used for washing of the farrowing unit:**			0.21			0.21	χ2
Manual washing	30 (71)	47 (90)		30 (71)	47 (90)		
Robot combined with manual washing	12 (29)	9 (17)		12 (29)	9 (17)		
Missing	0 (0)	(0)					
**Disinfection of the farrowing unit between batches:**			0.09			0.07	χ2
Yes	27 (64)	25 (45)		28 (67)	26 (46)		
No	14 (33)	29 (52)		14 (33)	30 (54)		
Missing	1 (2)	2 (4)					
**Maternal vaccination against NPD:**			0.02			0.02	Fisher's exact
Yes	39 (93)	43 (77)		40 (95)	43 (77)		
No	2 (5)	13 (23)		2 (5)	13 (23)		
Missing	1 (2)	0 (0)					
**Cross-fostering of piglets:**			1			1	Fisher's exact
Yes	41 (98)	55 (98)		55 (98)	55 (98)		
No	1 (2)	1 (2)		1 (2)	1 (2)		
Missing	0 (0)	0 (0)					
**Employment of nurse sows:**			0.01			0.01	χ2
Yes	24 (57)	17 (30)		24 (57)	17 (30)		
No	18 (43)	38 (68)		18 (43)	39 (70)		
Missing	0 (0)	1 (2)					

	Original data			Imputed data			
Variable [a]	NPD herds n (%)	Non-NPD herds n (%)	P-value	NPD herds n (%)	Non-NPD herds n (%)	P-value	Test
**Entry of sows to the farrowing unit (no. of days before farrowing):**			0.41			0.36	χ2
<4	13 (31)	15 (27)		13 (31)	15 (27)		
4-7	22 (52)	28 (50)		24 (57)	28 (50)		
>7	5 (12)	13 (23)		5 (12)	13 (23)		
Missing	2 (5)	0 (0)					
**Monitoring of farrowings:**			0.17			0.17	Fisher's exact
Only in special cases	3 (7)	11 (20)		3 (7)	11 (20)		
During daytime	35 (83)	38 (68)		35 (83)	38 (68)		
During day and night	4 (10)	7 (13)		4 (10)	7 (13)		
Missing	0 (0)	(0)					
**Criteria used for clinical assessment of post-partum dysgalactia syndrome (PPDS):**			0.44			0.42	Fisher's exact
Fever/inappetence	6 (14)	10 (18)		6 (14)	10 (18)		
Fever/inappetence + additional clinical signs	23 (55)	28 (50)		24 (57)	28 (50)		
Defined fever (>39°C)	11 (26)	12 (21)		11 (26)	12 (21)		
Other	1 (2)	6 (11)		1 (2)	6 (11)		
Missing	1 (2)	0 (0)					

Variable [a]	Original data			Imputed data			
	NPD herds n (%)	Non-NPD herds n (%)	P-value	NPD herds n (%)	Non-NPD herds n (%)	P-value	Test
**First choice of antimicrobial to treat PPDS:**			0.31			0.36	Fisher's exact
Trimethoprim/ sulphonamide	34 (81)	39 (70)		34 (81)	41 (73)		
Penicillin	7 (17)	9 (16)		7 (17)	9 (16)		
Other	1 (2)	6 (11)		1 (2)	6 (11)		
Missing	0 (0)	2 (4)					
**Efforts made to save weak-born piglets:**			<0.01			<0.01	χ2
None/some	18 (43)	43 (77)		18 (43)	43 (77)		
Moderate/ambitious	24 (57)	13 (23)		24 (57)	13 (23)		
Missing	0 (0)	(0)					
**Type of supplemental heating in the creep area:**			0.14			0.19	Fisher's exact
Heat lamp	10 (24)	24 (43)		11 (26)	24 (43)		
Floor heating	4 (10)	6 (10)		4 (10)	6 (10)		
Lamp and floor heating	27 (64)	25 (45)		27 (64)	25 (45)		
Other	0 (0)	1 (2)		0 (0)	1 (2)		
Missing	1 (2)	0 (0)					
**Control of the temperature in the creep area of the farrowing pen:**			0.41			0.46	χ2
Yes	11 (26)	10 (18)		11 (26)	10 (18)		
No	29 (69)	45 (80)		31 (74)	46 (82)		
Missing	2 (5)	1 (2)					

	Original data			Imputed data			
**Variable** [a]	**NPD herds n (%)**	**Non-NPD herds n (%)**	**P-value**	**NPD herds n (%)**	**Non-NPD herds n (%)**	**P-value**	**Test**
**Desired temperature in the farrowing unit:**			0.24			0.26 [e]	Fisher's exact
<18	4 (10)	12 (21)		4 (10)	12 (21)		
18-20	26 (62)	27 (48)		26 (62)	33 (59)		
>20	10 (24)	7 (13)		10 (24)	7 (13)		
Variable	2 (5)	3 (5)		2 (5)	4 (7)		
Missing	0 (0)	7 (13)					
**Covered creep area:**			1			1	Fisher's exact
Yes	10 (24)	14 (25)		10 (24)	14 (25)		
No	31 (74)	40 (71)		31 (74)	41 (73)		
Not in all pens	1 (2)	1 (2)		1 (2)	1 (2)		
Missing	0 (0)	1 (2)					
**Criteria used for clinical assessment of NPD:**			0.51			0.51	Fisher's exact
Diarrhoea	7 (17)	11 (20)		7 (17)	11 (20)		
Diarrhoea and depressed general condition	32 (76)	44 (79)		32 (76)	44 (79)		
Other	3 (7)	1 (2)		3 (7)	1 (2)		
Missing	0 (0)	0 (0)		0 (0)	0 (0)		

[a] *Respondents were informed to base their answers on an average farrowing batch during the last 12 months.*

[b] *These variables refer to the first week after farrowing.*
