# Supplementary material for: Farm characteristics and management routines related to neonatal porcine diarrhoea: a survey among Swedish piglet producers
Source: Acta Vet Scand. 2016 Nov 10;58:77. doi: 10.1186/s13028-016-0261-0 (PMC5103491; doi:10.1186/s13028-016-0261-0)
Supplement: Supplementary file 2 — Additional file 2: Table S2. Associations with herd size. Descriptive data and univariable associations between herd size (<200 and >200 sows in production) and explanatory variables selected for regression analyses. [file 13028_2016_261_MOESM2_ESM.pdf]

Additional file 2. Descriptive data and univariable associations between variables selected for regression analyses and herd size in 64 herds with <200 sows in production (SIP) and 34 herds with >200 SIP

Variable [a]	<200 SIP	>200 SIP	*P*-value[b]
	n (%)	n (%)	
**Building or renovation of stables used as farrowing units:**			<0.01[c]
Earlier than 1990	14 (22)	0 (0)	
1990-2000	21 (33)	4 (12)	
Varies between units	12 (19)	10 (29)	
Later than 2000	17 (27)	20 (59)	
**Recording of production results:**			<0.01[c]
Yes	50 (78)	34 (100)	
No	14 (22)	0 (0)	
**Level of gilt recruitment:**			<0.01[c]
<30%	23 (36)	4 (12)	
30-40%	19 (30)	10 (29)	
>40%	11 (17)	16 (47)	
Unknown	11 (17)	4 (12)	
**Manual cleaning of the farrowing unit (times/day)** [c]**:**			<0.01[d]
0-1	28 (44)	25 (74)	
2-3	36 (56)	9 (26)	
**Washing of the farrowing unit between batches:**			<0.01[c]
Always	35 (55)	32 (94)	
During summer	13 (20)	1 (3)	
No	16 (25)	1 (3)	
**Disinfection of the farrowing unit between batches:**			0.11[d]
Yes	31 (48)	23 (68)	
No	33 (52)	11 (32)	
**Maternal vaccination against NPD:**			<0.01[c]
Yes	49 (77)	34 (100)	
No	15 (23)	0 (0)	
**Employment of nurse sows:**			<0.01[d]
Yes	18 (28)	23 (68)	
No	46 (72)	11 (32)	

**Monitoring of farrowings:**			0.5 [c]
Only if indicated	11 (17)	3 (9)	
During daytime	45 (70)	28 (82)	
During day and night	8 (13)	3 (9)	
**Efforts made to save weak-born piglets:**			0.04[d]
None/some	45 (70)	16 (47)	
Moderate/ambitious	19 (30)	18 (53)	
**Type of supplemental heating in the creep area:**			0.02[c]
Heat lamp	29 (45)	6 (18)	
Floor heating	6 (9)	4 (12)	
Lamp and floor heating	28 (44)	24 (71)	
Other	1 (2)	0 (0)	

[a] *Respondents were requested to base their answers on an average farrowing batch during the last 12 months.*

[b] *P- value of the entire variable*

[c] *Fisher's exact test*

[d] *χ2- test*
